# An *In vivo* study: Adjuvant activity of poly-n-vinyl-2-pyrrolidone-co-acrylic acid on immune responses against Melanoma synthetic peptide

**DOI:** 10.1080/21655979.2017.1373529

**Published:** 2017-09-28

**Authors:** Kadriye Kızılbey, Banu Mansuroğlu, Serap Derman, Zeynep Mustafaeva Akdeste

**Affiliations:** a Biomedical Engineering Department, İstanbul Yeni Yüzyıl University, Yılanlı Ayazma Caddesi, Zeytinburnu-İstanbul, Turkey; b Department of Molecular Biology and Genetics, Yıldız Technical University, Davutpaşa Caddesi, Esenler, İstanbul, Turkey; c Department of Bioengineering, Yıldız Technical University, Davutpaşa Caddesi, Esenler, İstanbul, Turkey

**Keywords:** cancer, conjugate, complex, ELISA, melanoma, T-Cell epitopes, P(VP-*co*-AA)

## Abstract

Peptides have been studied as an important class of components in medicine to control many major diseases with vaccination. Polymers as adjuvants are capable of enhancing the vaccine potential against various diseases by improving the delivery of antigens, and they reduce the booster doses of vaccines. In brief, polymers are promising candidates for peptide-based vaccine delivery platforms. The purpose of the present study was to create a possible alternative approach in the treatment of malignant melanoma and/or to prevent metastasis of melanoma. The study was designed as both an experimental and an in vivo study. We prepared a complex and covalent conjugate of MAGE-3 121–134 (L-L-K-Y-R-A-R-E-P-V-T-K-A-E) T-cell epitope as a vaccine candidate for melanoma. These conjugates were able to generate an immune response in mice after a single immunization, without the help of any external adjuvant. The peptide-polymer complexes activated the immune system in the best way and formed the highest antigen specific immune response. These results indicate the adjuvant activity of Poly(N-vinyl-2- pyrrolidone-co-acrylic acid) [P(VP-co-AA)] and the potential use of P(VP-coAA)-peptide based vaccine prototypes for future melanoma cancer vaccine formulations.

## Introduction

Malignant melanoma incidences are rising faster than any other form of cancer. Over 75% of all skin cancer deaths are from this kind of cancer, despite the fact that it represents only 10% of all skin malignancies.^1,2^ Currently representing 2% of all new cases of cancer and 1% of all cancer deaths, the incidence of melanoma continues to rise in Caucasians at a rate of approximately 5% per year. This rate is higher than any other solid tumor except lung cancer in women. In the United Kingdom (UK), there are 6000 new cases every year resulting in 2000 deaths; in Australia and New Zealand, it is 10 times more common. In the United States of America (USA), the corresponding numbers are 32100 new cases and 7200 deaths^3^ every year.

Due to their high tumor specificity as well as being found in many tumors, MAGE-1, 2, and 6 antigen epitopes have particular importance for cancer immunotherapy.^4-6^ The melanoma-associated antigen (MAGE) gene was discovered by Van der Bruggen et al. in 1991 and isolated from a MZ-2 human melanoma cell line. MAGE proteins contain a super family of more than 60 genes in humans.^7-10^ MAGE family members have important physiological and pathological roles in embryogenesis, germ cell genesis and apoptosis. The MAGE family is divided into two groups that included Type I-MAGE-I (MAGE-A, MAGE-B and MAGE-C) and Type II-MAGE-II (MAGE-D).^9,10^

In contrast to members of the MAGE-I group, the MAGE-II member is not related to cancer. MAGE-I proteins are normally expressed in the testes, trophoblast, and placenta^9,11^ and are important targets for cancer immunotherapy and have been vital in some clinical trials treating gastrointestinal carcinoma, esophageal carcinoma and pulmonary carcinoma. Furthermore, investigations into their expression have proven useful for determining cancer biomarkers in the early diagnosis of cancers. Additionally, these proteins are named as cancer/ testis (CT) antigen and tumor-specific antigen.^8,9^ For this reason, MAGE-I proteins and peptides are considered as candidates for the development of cancer vaccines.^7,9^ Complexes with human leukocyte antigens could be recognized by autologous T-lymphocytes.^8^

As metastatic melanoma is largely chemo-resistant,^12,13^ new treatments such as inducing specific antibody production,^14^ expression of cytokines,^15,16^ and intra-tumorous T-lymphocytic infiltrates^17^ that lead to complete long-term remissions of cutaneous melanoma metastases are of importance.

Recently, peptides have been investigated as an important class of components in medicine to control many major diseases with vaccination. Synthetic peptides are not sufficiently large or complex by themselves to induce the immune system. Therefore, peptides have been used in combination with different adjuvant systems such as synthetic polymers.^18^

Polymers induce the vaccine potential of antigens by improving their delivery and reducing the booster doses of vaccines against several antigenic diseases as an adjuvant. In brief, polymers are promising candidates for peptide-based vaccine delivery platforms. Rafikov and colleagues showed that the copolymers of N-vinyl-2-pyrrolidone with acrylic acid (P(VP-*co*-AA)) have a pronounced immune-stimulating effect and could be used as an adjuvant for the immunological system because of their biocompatibility.^19^

The purpose of this study was to create a possible alternative approach in the treatment of malignant melanoma and/or in the prevention of the metastasis of melanoma. Therefore, we synthesized the MAGE-3 121–134 (L-L-K-Y-R-A-R-E-P-V-T-K-A-E) peptide sequence by the microwave assisted solid phase peptide synthesis (SPPS) method. This peptide was chemically conjugated using two different methods and physically mixed with P(VP-*co*-AA) polymer. Activation of the immune system could not be achieved using the peptide alone. We evaluated the efficacy of P(VP-co-AA) copolymer as a carrier to form P(VP-co-AA)-peptide based vaccines prototypes in the activation of the immune system against the melanoma cancer-associated antigenic MAGE peptide.

## Materials and methods

### Reagents

Preloaded Wang resins, amino acids and coupling reagents purchased from NovaBiochem (Nottingham, England). The other chemicals for peptide synthesis were purchased from Sigma Aldrich (Carlsbad, CA, USA). Acrylic acid (Aldrich, Seelze, Germany), N-vinyl-2-pyrrolidone, ethyl acetate, benzoyl peroxide and cobalt naphthenate (Fluka, Seelze, Germany), Tetrahydrofuran (THF), trifluoroacetic acid (TFA), Thioanisole (Riedel-de Haen, Hannover, Germany), 1-ethyl-3-(3-dimethylaminopropyl) carbodiimide hydrochloride (Sigma, Carlsbad, CA, USA) were of analytical grade. NaH_2_PO_4_, Na_2_H-PO_4._7H_2_O, NaCl were obtained from Fluka (Seelze, Germany) and NaN_3_ was from Applichem (Darmstadt, Germany).

#### Synthesis of MAGE-3 121–134 Peptide Sequence

MAGE-3 121–134 (L-L-K-Y-R-A-R-E-P-V-T-K-A-E) antigenic peptide sequence was synthesized by the SPPS method in dimethylformamide (DMF, Sigma Aldrich, Carlsbad, CA, USA) media. O-Benzotriazole-N,N,N’,N’-tetramethyl-uronium-hexafluorophosphate/N-Hydroxybenzotriazole (HBTU/ HOBt) were used as activators and N,N-Diisopropylethylamine / N-Methyl-2-pyrrolidone (DIEA/NMP) were used as activator bases (Sigma Aldrich, Carlsbad, CA, USA).^20,21^ The peptide was cleaved from resin using a cleavage cocktail prepared with TFA/ Thioanisole/ 1,2-Ethane dithiol (EDT, Sigma Aldrich, Carlsbad, CA, USA)/ Water: 90/ 5/ 2,5/ 2,5 (v/v). Cold (−20°C), and ether was used for precipitation. After the centrifugation, precipitated peptide was dried in a vacuum.

#### Synthesis of Poly(N-vinyl-2-pyrrolidone-*co*-acrylic acid)

A 2:1 monomer ratio of P(VP-*co*-AA) was synthesized by the free radical polymerization method and characterized^22^ in our previous studies. THF was used in the polymerization of glacial acrylic acid (Fluka, Seelze, Germany) and N-vinyl-2-pyrrolidone (Fluka, Seelze, Germany) at 70°C under nitrogen for 3 hours. Benzoyl peroxide (Sigma Aldrich, Carlsbad, CA, USA) and cobalt naphthenate (Sigma Aldrich, Carlsbad, CA, USA) were used as radical initiators. The average molecular weight of the synthesized polymer was calculated as 120 kDa with a polydispersity Mw/Mn = 2.701.

#### Synthesis of Peptide-Polymer Complexes and Covalent Conjugates

In this study, 3 different methods were used to prepare conjugates and complexes. One method is the complex formation method and the others involve the synthesis of peptide-P(VP-*co*-AA) conjugates.

In Method I, P(VP-*co*-AA) and peptide were covalently conjugated in a two-step reaction using water-soluble carbodiimide as a cross-linker agent;^23-25^ 50 mM PBS (pH 7.0) was used to dissolve both P(VP-*co*-AA) and peptide separately. The carboxyl group in the polymer chain was activated in pH 5.0 water by EDC in a 4:1 molar ratio of EDC:AA. The pH of the polymer solution was set to 5.0, and EDC was added. The peptide solution was added to the active polymer solution after it was stirred for 1 h at room temperature. After adding the peptide, the polymer-peptide mixture solution was stirred for an additional 12 h at 4°C, and the pH was adjusted to 7.0 with 1 M NaOH. The *O*-acylisourea intermediate was removed by dialysis.

In Method II, conjugates of P(VP-*co*-AA) and peptide were synthesized in organic media using microwave energy. The peptide and polymer were dissolved in dimethylformamide (DMF). Tetramethyluronium hexafluorophosphate (HBTU) and 1-hydroxybenzotriazole (HOBt) were added into the polymer solution for the activation of polymer. N,N-Diisopropylethylamine/N-Mehtylpyrrolidone (DIEA/ NMP) was added into the peptide solution for the activation of peptide. Then, the activated peptide was added to the polymer solution for conjugation. Conjugation occurred in 15 minutes under 300 W of microwave energy (Milestone Microsynth). After the conjugation, 4°C ethyl acetate was added to the sample to precipitate the peptide-P(VP-co-AA) conjugate.

Method III involved complex formation of P(VP-*co*-AA) and peptide. Both peptide and copolymer were dissolved in 50 mM PBS at pH 7.0 separately. Complexes of P(VP-*co*-AA) and peptide were prepared by physical mixture of solvents with intermolecular interactions.^26^

All complexes and conjugates were centrifuged with Sartorious VIVASPIN (10.000 MW) (Göttingen, Germany) polyethersulfon (PES) membrane tubes for 6 minutes at +4°C, 6000 rpm. After centrifugation, the upper part and pre-purification samples were analyzed in HPLC (Shimadzu, Cintech IV, Singapore). After characterization and purification, complexes and conjugates were lyophilized at −80°C. The powdered samples were dissolved in 0.154 M NaCl^27^ and injected into mice.

#### Gel permeation chromatography analysis of complexes and conjugates

Gel permeation chromatography was used for analyses of water-soluble peptide-polymer complexes and conjugates with Shim-Pack Diol-300 (7.9 mm ID × 50 cm, 24°C) at a 1.0 ml/min flow rate. A Shimadzu SPD-10 AV VP model UV-VIS detector was used for monitoring of the elution at 280 nm. A 20 µl sample was automatically injected for analysis, and all solutions were filtered through 0.45-µm Sartorius RC-membrane filters before injection. The column was calibrated with Aldolase (150 kDa), Human Serum Albumin (66 kDa), Carbonic Anhydrase of Bovine Erythrocytes (29 kDa) standards (Sigma Aldrich (Carlsbad, CA, USA).^28^

In the mobile phase, PBS (0.05 M phosphate and 0.15 M sodium chloride) at pH 7.0, was filtered through a 0.45-µm Sartorius RC cellulose nitrate filter and degassed before use.

#### Immunization

Twenty-four 8 week old male mice (Balb-c) (28-32 g) were used in all experiments. Clean, sterile, polypropylene cages under a controlled photoperiod (12 h light/dark cycle) and humidity (55-60%) were used in the housing of the animals. The ethical treatment of experimental animal guidelines was followed in all experimental protocols. The appropriate animal ethical committee certified this study and limited the animal number to 4 for each group. The mice were divided into 6 groups randomly with 4 animals in each group. After characterization and purification, samples were intraperitoneally injected under ether anesthesia in one-shot immunization into 6 groups on the same day. Only Group 5 received a second injection as a booster immunization on the 49^th^ day after the first injection (Table 1).

**Table 1.** Groups and injected samples' contents of conjugate and physical complexes.

Groups	Injected sample	Injection volume (ml)	Synthesis Method	Peptide amount (mg)/ 0.5 ml	Balb/c Mice Number in each group
1	BSA-Peptide conjugate	0.5	I	1	4
2	P(VP-co-AA)-Peptide conjugate	0.5	I	1	4
3	P(VP-co-AA)-Peptide conjugate	0.5	II	1	4
4	P(VP-co-AA)-peptide complex	0.5	III	0.5	4
5	*Multi Injection*:				4
	*I Injection*:	0.5	III	1	
	P(VP-co-AA)-peptide complex				
	*II Injection*:	0.5	III	0.5	
	P(VP-co-AA)-peptide complex				
6	Control 0.154 M NaCl	0.5	—	0	4

BSA: Bovine serum albumin; P(VP-co-AA): Poly(N-vinyl-2-pyrrolidone-co-acrylic acid); NaCl: Sodium Chloride.

#### Elisa method

The antibodies were measured in tail-vein blood samples from the immunized mice weekly at 10 weeks; 20 μl blood samples were collected in microfuge tubes containing sodium citrate. The tubes were centrifuged at 2500 rpm for 3 min to remove the blood cells, and the plasma samples were tested by the ELISA method. Serum physiologic injected mice serums were used as controls.

The indirect enzyme-linked immunosorbent assay (ELISA)^29,30^ was used for the screening of the serum for anti-peptide activity; 96 well ELISA plates (Greiner) were coated with 100 ng BSA-peptide conjugates. Another plate was coated only with BSA as a control to see the Ab titer against BSA for the 1^st^ group. All plates were kept at +4°C overnight. The plates were washed two times with washing buffer (0.005% tween-20 in 0.5 M PBS). After washing, 200 μl 0.2% milk powder in PBS was added to the wells and incubated for 1 h at 37°C. Then, the procedure was followed by washing as above. Each serum was diluted with dilution buffer to 1:50 and 1:100; 100 μl diluted serum samples were added to plate. The plate was kept at +4°C for two hours and washed as above. After washing, 100 μl alkaline phosphatase conjugate of polyvalent goat-antimouse Ig (SIGMA) in 1:750 dilution buffer (PBS) was added to each well and incubated for 1 h at 37°C. After incubation, the plate was washed five times with washing buffer. Then, 0.1% para-nitrophenyl phosphate (PNFP) substrate buffer was added, and the plate was held in a dark room for 45 minutes. Finally, the absorption of the plate was read at 405 nm, and the antibody amount was determined.^27,29,31^

#### Statistical analysis

The immunization results were performed with t-test (Microsoft Office, Northern California, USA) statistical methods. There was a significant difference between groups but no indication regarding which group caused the difference. For to this reason, the groups were compared in pairs using in pair comparison t-tests. Means were compared and significances were controlled. T values calculated of all the experimental groups for each week were as p > 0.05. As a result, the correlation was important and not accidental.

## Results

This study was intended to examine the adjuvant effect of P(VP-*co-*AA) polymer in the covalent conjugation or physical complexation with the MAGE 121–134 peptide. First, the MAGE 121–134 peptide (Mw: 1860 Da) and P(VP-*co*-AA) polymer (Mw:120.000 Da) were synthesized. Then, peptide-polymer complexes and conjugates were analyzed by Gel Permeation Chromatography method and used for immnunization of the Balb-c mice.

### Gel permeation analysis of complexes and conjugates

Synthesized MAGE 123–134 peptide was conjugated to P(VP-*co*-AA) polymer with carbodiimide conjugation procedure. Conjugates and complexes occurrence and characterization were determined by HPLC.

**Figure 1.**
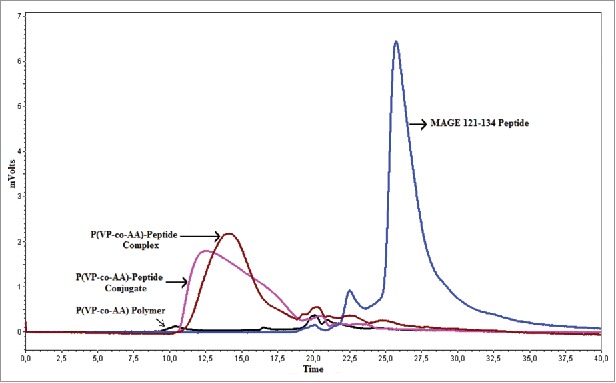
Comparative HPLC chromatogram of P(VP-*co*-AA)-Peptide complex, P(VP-*co*-AA)-Peptide conjugates, MAGE 121–134 peptide and P(VP-*co*-AA) polymer.

P(VP-*co*-AA), MAGE 123–134 peptide, their complexes and their conjugates are labeled in the comparative chromatogram, which is shown in Fig. 1. In addition to the major MAGE 123–134 peptide peak appearing around the 26th minute, the copolymer displayed a baseline characteristic as shown in Fig. 1. After the conjugation and the complexation reactions, the UV detector detected new peaks between 10.5 and 19 minutes in samples. This finding indicates complex formation with negative groups of polymers and positive groups of peptides in complex formation and conjugation between carboxyl group of polymer and amino group of peptide in conjugation reactions.

### Immunology results

**Figure 2.**
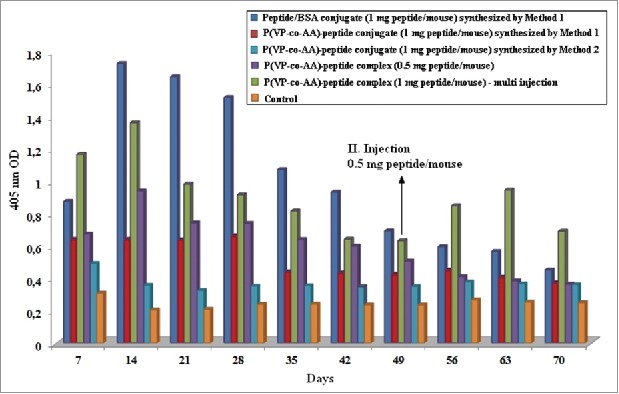
Comparison of the amount of antibody determined from all groups by ELISA in mice sera at 7 day intervals for 70 days.

There are 6 immunization groups consisting of BSA-peptide conjugate, P(VP*-co-*AA)-peptide conjugate, microwave-assisted P(VP-*co*-AA)-peptide conjugate, P(VP-*co*-AA)-peptide complex and control group. Free peptide was investigated in our previous studies, and because of the small molecule size and fast degradation in biological systems, it was observed that free peptide (standard drug) does not produce antibody answer as indicated in the literature.^32-36^ Because of this reason, a free peptide group was not studied in the present work. Additionally, the group containing physiological NaCl was used as a control. In addition to these groups, Group 5 is named as multiple injection group and has two injections in total. The first injection is P(VP-*co*-AA)-peptide complex with 1 mg peptide/0.5 ml dose, the second injection is P(VP-*co*-AA)-peptide complex with 0.5 mg peptide/0.5 ml dose as the memorial stroke. Fig. 2 shows the kinetics of a specifically formed antibody against P(VP-*co*-AA)-peptide complex and conjugates. Antibody titrations were calculated from mouse sera in 7-day intervals for 70 days by the indirect ELISA method^27,29,30,37-41^ from intraperitoneally injected BALB/c mice.

Injections of mice with BSA-peptide conjugate were determined as the first experimental group. According to Method I in the presence of EDC, BSA-peptide conjugate (Group 1) containing 1 mg peptide/mouse were synthesized and vaccinated in a single dose to BALB/c mice. A high level of specific antibody formation in the mice serum was observed against this conjugate with single dose immunization compared with control and all other test groups. The amount of antibody began to decline after reaching a maximum on the 14^th^ day. Similar to other studies, when compared with the control group a significantly high specific antibody amount was seen against BSA-peptide conjugate even on the 70^th^ day after the injection.^38,41-46^

Injections of mice with P(VP-*co*-AA)-peptide conjugate were determined as the second experimental group. According to Method I in the presence of EDC, P(VP-*co*-AA)-peptide conjugate (Group 2) containing 1 mg peptide/mouse were synthesized and vaccinated in a single dose to BALB/c mice. A low level of specific antibody formation in mice sera was observed against this conjugate with single dose immunization compared with the other test groups. The amount of antibody reached the maximum on the 14^th^ day. After reaching the maximum on the 14^th^ day, the antibody values remained the same until day 28 and began to decline after the 28^th^ day.

Injections of mice with another P(VP-*co*-AA)-peptide conjugate were determined as the third experimental group. According to Method II using microwave energy in organic media, P(VP-*co*-AA)-peptide conjugate (Group 3) containing 1 mg peptide/mouse were synthesized and vaccinated in a single dose to BALB/c mice. The lowest level of specific antibody formation in mice sera was observed against this conjugate with single dose immunization compared with all other test groups. ELISA assays showed very low levels of antibody formation in the same manner for 70 days.

**Figure 3.**
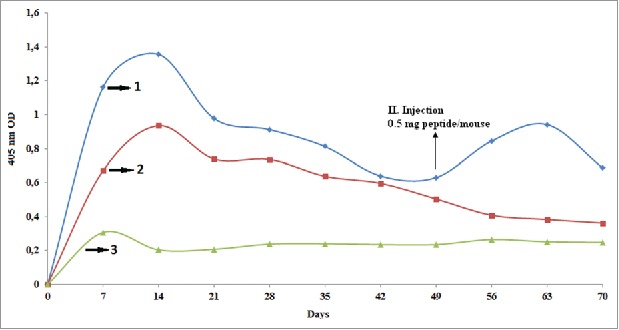
Comparison of the amount of antibody determined by ELISA in mice sera at 7 day intervals for 70 days against: (1) P(VP-co-AA)-peptide complexes (1 mg/mouse in 0.5 ml), (2) P(VP-co-AA)-peptide complexes (0.5 mg/mouse in 0.5 ml) and (3) control groups.

Parallel to conjugates, P(VP-*co*-AA)-peptide complexes that were prepared by Method III were determined as Group 4 and 5 for injections. As a result of physical interactions, P(VP-*co*-AA)-peptide complexes (Group 4 and 5) containing 0.5 mg and 1 mg peptide/mouse were prepared and vaccinated in variable injection doses to BALB/c mice. Specific antibody formation in mice sera was observed in Group 4 against P(VP-*co*-AA)-peptide complex containing 0.5 mg peptide/mouse with single dose immunization compared with P(VP-*co*-AA)-peptide conjugate test groups. The amount of antibody began to decline after reaching a maximum on the 14^th^ day (Fig. 3).

Specific antibody formation in mice sera was highly observed in Group 5 against P(VP-*co*-AA)-peptide complex containing 1 mg peptide/mouse at the first dose immunization compared with P(VP-*co*-AA)-peptide conjugate test groups except the peptide-BSA conjugate group. In comparison to Group 4, twice as much of an increase was observed in the antibody amount when the concentration of peptide in the first injection was increased two-fold at group 5. This result was because of the difference in the peptide concentrations in the complexes. As similar to group 4′s antibody titration, the amount of antibody began to decline after reaching a maximum on the 14^th^ day (Fig. 3).

Due to the similarity of the two groups with each other, the decline of antibody titration is assumed. After the realization of this decline, a second injection was performed on the 49^th^ day. A second injection of complex containing 0.5 mg of peptide per mouse (half dose) to Group 5 was applied. However, because it was a half-dose injection, it had memory effect as expected. After the second injection, antibody titration increased until reaching its second maximum on the 63^rd^ day but then began to decline again. There was a high degree of specific antibody formation against P(VP-*co*-AA)-peptide complex even compared to control group in the 70^th^ day after the first injection. Compared to all groups except Group 1, a higher antibody level was observed.

**Figure 4.**
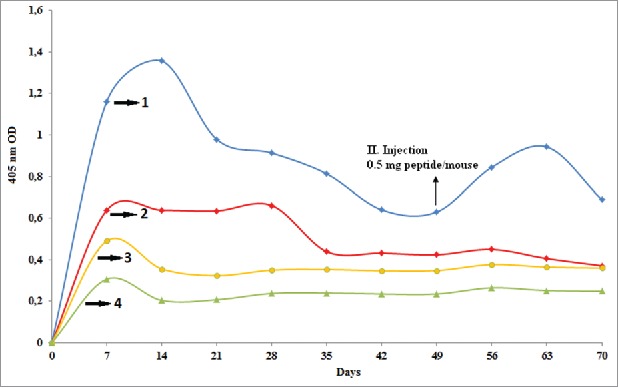
Comparison of the amount of antibody determined by ELISA in mice sera in 7 day intervals for 70 days against: (1) Peptide-P(VP-co-AA) complex (1 mg/0.5 ml) prepared by Method III (Group 5: multiple injection group), (2) Peptide-P(VP-co-AA) conjugate (1 mg/0.5 ml) synthesized by Method I (Group 2), (3) Peptide-P(VP-co-AA) conjugate (1 mg/0.5 ml) synthesized by Method II (Group 3), and (4) control groups.

Antibody titration in mice sera against P(VP-*co*-AA)-peptide physical complexes (Group 4 and Group 5) prepared to contain 0.5 mg and 1 mg of peptide per mouse were much higher than samples compared with P(VP-*co*-AA)-peptide conjugates synthesized according to Method I and II. However, the immune response against P(VP-*co*-AA)-peptide conjugates synthesized by Method I was higher than the immune response against P(VP-*co*-AA)-peptide conjugates synthesized by Method II (Fig. 4). As a result of these studies, it could be said that P(VP-*co*-AA)-peptide physical complexes induced immune responses more than P(VP-*co*-AA)-peptide conjugates.

## Discussion

Adjuvant immunotherapy is a potential therapeutic treatment for the improvement of results of early-stage non-small cell lung cancer. For example, MAGE-A3 is especially presented on the cell surface of cancer cells and considered to be correlated with an aggressive cancer phenotype. Therefore, it might be a promising target for immunotherapy.^47^ Some research groups have focused on the development of MAGE-A monoclonal antibodies (mAb). Picard et al. have worked on mAb and detected MAGE-A9 expression by immunohistochemistry. After this improvement, mAb, which could recognize an epitope located at the COOH terminus of the MAGE-A10 gene product, was produced. Researchers detected that MAGE-A10 was expressed in a high proportion of tumor cell in lung, skin and urothelial malignancies by using multitumor tissue microarray.^8^

Several in vitro studies indicated that MAGE proteins have an effect on p53 mediated apoptosis and cell proliferation. It was found that CT-X expression had a correlation with advanced and aggressive tumors. There have also been studies about MAGE genes that explain the relationship of their expression with improved prognosis and survival. In one study, human melanoma and ovarian samples were used, and both had frequent CT-X expression. According to their results, mutations in melanoma genes were monitored, and no mutations in genes of ovarian tumors were observed.^48^

Due to a lack of biological markers and specific treatment, lung cancers are the most compelling cancer type. Karimi and his colleagues researched melanoma-associated antigen-A gene expression in both tumorous and non-tumorous tissue. They focused on the early MAGE usage level for the prevention of lung cancer. Their aim was to examine the expression side of various MAGE genes in non-small-cell lung cancer tumorous tissue. They investigated tumorous and non-tumorous genes and found that the MAGE-A4 gene had the peak incidence density in all MAGE-A genes. Experiments revealed that MAGE-A proteins could be considered strong candidates as molecular tumor markers for early diagnosis.^49^

In our study, the first 121–134 of melanoma tumor Mage3 peptide sequence was synthesized by Merrifield Solid-State Peptide Synthesis chemical method. Biocompatible, biodegradable and nontoxic P(VP-*co*-AA) polyelectrolytes are selected to carry the antigenic peptide molecule in living systems. Then, complex and conjugate formations between the peptide and polyelectrolyte were tested. Complex formation occurred due to the electrostatic interactions of positively charged peptide (pI = 10) with negatively charged polymers in pH = 7 PBS solution. P(VP-*co*-AA)-peptide conjugates were synthesized by conventional covalent conjugation and microwave energy methods using carbodiimide cross-linkers in both methods. To understand the importance of conformational differences in immunological experiments, complexes were prepared and conjugates were synthesized in different ways. High titers of specific antibodies against peptide formation were observed in mice blood sera with peptides containing polymeric conjugates and complexes. In the literature, it is also said that low molecular weight synthetic peptide antigens should bind to a carrier molecule to obtain an immune response against these antigens.^33-36,38^

This study showed that the effectiveness of the peptide vaccine is related with the presence of T-epitopes in conjugates' and complexes' structures for the tumor-specific T-helper lymphocytes' activation.

In this article, it was observed that after an intraperitoneal injection of a single dose of peptide-BSA conjugate, the specific antibody response began to rise rapidly and then decreased after reaching the maximum on the 14^th^ day. T-cell epitopes, which were conjugated with BSA, showed high immunity in the literature.^38,41,42,44-46^ Dilgimen and colleagues^41^ had injected copolymer-BSA conjugate containing equal concentration solutions of BSA into mice. The immune response formation against low and high concentration of copolymer-BSA conjugates was studied. Antibody formation was triggered in a dose-dependent free BSA injection. Very low antibody formation was reported against low copolymer-BSA conjugate solutions. The immunological activity continued for 50 days and was observed against concentrate copolymer-BSA solutions after reaching a maximum on the 7th day.

In immunological studies as mentioned in other studies, it was believed that the conformations of complexes and conjugates were the reason for the low immune responses in Group 3 and 2. If we examine a further aspect, the levels of specific antibody in Group 5 against P(VP-*co*-AA)-peptide complex is two times greater than the antibody formation in Group 4 against the complex. This difference was because of the difference in the peptide concentrations in complexes.

When the physical mixture and conjugates of polyelectrolytes was compared with melanoma peptide, the highest immune response formed against the peptide-P (VP-*co*-AA) physical complex occurring with electrostatic interaction. The results obtained from the study showed that high immune response was related with the conformation of the complex. The peptide sequences in a complex remained deficit and interacted easily with the blood plasma proteins and cell surface proteins.

The immune responses of conjugates synthesized by Method I (classical conjugation) according to the presence of EDC and Method II according to the microwave energy were compared. The immune response of conjugates synthesized according to Method I was higher than the other method. This was because the structure of conjugates synthesized by microwave method had more compact structures than those synthesized in the classical way. This hypothesis was approved by Z-Ave (mean particle size) values of conjugates synthesized by the microwave method and conventional method obtained as 30.67 nm and 42.34 nm, respectively.

Consequently, the complexes formed as a result of electrostatic interaction with the physical mixture activated the immune system in the best way and formed the highest antigen specific immune response. In this article, obtaining a high immune response against melanoma peptide is an important step towards achieving specific antibodies for the development of various protective therapies against melanoma and for the diagnosis of melanoma tumors.
